# Structural and physical properties of antibacterial Ag-doped nano-hydroxyapatite synthesized at 100°C

**DOI:** 10.1186/1556-276X-6-613

**Published:** 2011-12-03

**Authors:** Carmen Steluta Ciobanu, Florian Massuyeau, Liliana Violeta Constantin, Daniela Predoi

**Affiliations:** 1 National Institute of Materials Physics, 105 bis Atomistilor, P.O. Box MG 07, 077125, Bucuresti-Magurele, Romania; 2 Institut des Matériaux-Jean Rouxel, 02 rue de la Houssinière BP 32 229, 44 322 Nantes, France; 3 Faculty of Physics, University of Bucharest, 405 Atomistilor, CP MG - 1, 077125, Bucuresti-Magurele, Romania

## Abstract

Synthesis of nanosized particle of Ag-doped hydroxyapatite with antibacterial properties is in the great interest in the development of new biomedical applications. In this article, we propose a method for synthesized the Ag-doped nanocrystalline hydroxyapatite. A silver-doped nanocrystalline hydroxyapatite was synthesized at 100°C in deionized water. Other phase or impurities were not observed. Silver-doped hydroxyapatite nanoparticles (Ag:HAp) were performed by setting the atomic ratio of Ag/[Ag + Ca] at 20% and [Ca + Ag]/P as 1.67. The X-ray diffraction studies demonstrate that powders made by co-precipitation at 100°C exhibit the apatite characteristics with good crystal structure and no new phase or impurity is found. The scanning electron microscopy (SEM) observations suggest that these materials present a little different morphology, which reveals a homogeneous aspect of the synthesized particles for all samples. The presence of calcium (Ca), phosphor (P), oxygen (O), and silver (Ag) in the Ag:HAp is confirmed by energy dispersive X-ray (EDAX) analysis. FT-IR and FT-Raman spectroscopies revealed that the presence of the various vibrational modes corresponds to phosphates and hydroxyl groups. The strain of *Staphylococcus aureus *was used to evaluate the antibacterial activity of the Ca_10-*x*_Ag*_x_*(PO4)6(OH)2 (*x *= 0 and 0.2). *In vitro *bacterial adhesion study indicated a significant difference between HAp (*x *= 0) and Ag:HAp (*x *= 0.2). The Ag:Hap nanopowder showed higher inhibition.

## 1. Introduction

Inorganic biomaterials based on calcium orthophosphate have their wide range of applications in medicine [1-4]. Among them, synthetic hydroxyapatite (HAP, Ca_10_(PO_4_)_6_(OH)_2_) is the most promising because of its biocompatibility, bioactivity, and osteoconductivity. Hydroxyapatite has been used to fill a wide range of bony defects in orthopedic and maxillofacial surgeries and dentistry [5-8]. It has also been widely used as a coating for metallic prostheses to improve their biological properties [9-11]. In recent years, the use of inorganic antibacterial agents has attracted interest for control of microbes. The key advantages of inorganic antibacterial agents are improved safety and stability [12-14]. The most antibacterial inorganic materials are the ceramics immobilizing antibacterial metals, such as silver and copper. Hydroxyapatite (HAp) has widely been used for bone repair and substitute because of its good biocompatibility, and the cation exchange rate of HAp is very high with silver ions. Silver, known as a disinfectant for many years, has a broad spectrum of antibacterial activity and exhibits low toxicity toward mammalian cells [12]. The most common technique to incorporate Ag into HAp coatings is via an ion exchange method, in which the Ca ions in HAp are replaced by Ag ions while dipping the HAp coatings into AgNO_3 _for a period of time [15,16]. The limitation of the ion exchange method is that Ag will reside mostly on the outer surface of the coating and will be quickly depleted *in vivo/in vitro *without long-term antibacterial effect. In order to achieve the continuous release of Ag, HAp coatings doped with Ag through the entire thickness have been developed using sol-gel [17,18], co-sputtering [19,20], and thermal or cold spraying [21,22]. Although Ag in small percentages can have an antibacterial effect, larger amounts can be toxic [18], and therefore optimization of the Ag concentration in the coating is critical to guarantee an optimum antibacterial effect without cytotoxicity.

From the view point of biomedical engineering, the element silver is well known for its broad spectrum antibacterial effect at very low concentrations [23], and it possesses many advantages, such as good antibacterial ability, excellent biocompatibility, and satisfactory stability [24,25]. The scientific literature points to the wide use of silver in numerous applications. It is well established that silver nanoparticles are known for their strong antibacterial effects for a wide array of organisms (e.g., viruses, bacteria, fungi) [26]. Therefore, silver nanoparticles are widely used in medical devices and supplies such as wound dressings, scaffold, skin donation, recipient sites, and sterilized materials in hospitals, medical catheters, contraceptive devices, surgical instruments, bone prostheses, artificial teeth, and bone coating. One can also observe their wide use in consumer products such as cosmetics, lotions, creams, toothpastes, laundry detergents, soaps, surface cleaners, room sprays, toys, antimicrobial paints, home appliances (e.g., washing machines, air, and water filters), automotive upholstery, shoe insoles, brooms, food storage containers, and textiles [27-30].

Previous studies have focused on preparation and characterization of silver nanoparticles (AgNPs) [31]. The exact antibacterial action of AgNPs is not completely understood [32]. On the other hand in the literature, the studies on the preparation and characterization of the silver-doped hydroxyapatite powders are almost absent. The antibacterial studies on the Ag:HAp nanopowder are not presented, too.

In this article, we propose a method for synthesized the nanocrystalline hydroxyapatite doped with Ag at 100°C. Preparation of Ag-doped hydroxyapatite by co-precipitation method at 100°C has several advantages over other techniques. Specifically, it can generate highly crystalline nanopowder Ag:HAp. The Ag:HAp nanocrystalline powders will be used for implantable medical devices. Ag-doped nanocrystalline hydroxyapatite powders are obtained. Other phase or impurities were not observed. The Ca_10-*x*_Ag*_x_*(PO_4_)_6_(OH)_2 _with *x *= 0 and 0.2 was synthesized by co-precipitation method at 100°C. The Ca_10-*x*_Ag*_x_*(PO_4_)_6_(OH)_2 _with *x *= 0.2 was synthesized by co-precipitation method at 100°C mixing AgNO3, Ca(NO_3_)_2 _· 4H_2_O, and (NH_4_)_2_HPO_4 _in deionized water. The structure, morphology, vibrational, and optical properties of the obtained samples were systematically characterized by X-ray diffraction (XRD), scanning electron microscopy (SEM), transmission electron microscopy (TEM), Fourier transform infrared (FT-IR), and FT-Raman spectroscopies. For reveal the presence of the silver in the Ag:HAp (*x *= 0. 2) nanopowder, the X-ray photoelectron spectroscopy (XPS) results are presented, too. In addition, the antibacterial activity of the Ca_10-*x*_Ag*_x_*(PO_4_)_6_(OH)_2 _with *x *= 0 and 0.2 is studied.

## 2. Experimental procedure

### 2.1. Sample preparation

All the reagents for synthesis including ammonium dihydrogen phosphate [(NH_4_)_2_HPO_4_], calcium nitrate [Ca(NO_3_)_2 _· 4H_2_O], and silver nitrate (AgNO_3_) (Alpha Aesare) were purchased and used without further purification.

The Ca_10-*x*_Ag*_x_*(PO_4_)_6_(OH)_2_, with *x *= 0 (HAp), ceramic powder was prepared (Ca/P molar ratio--1:67) using Ca(NO_3_)_2_·4H_2_O and (NH_4_)_2_HPO_4 _by co-precipitation. A designed amount of ammonium dihydrogen phosphate [(NH_4_)_2_HPO_4_] was dissolved in deionized water to form a 0.5-mol/L solution. A designed amount of calcium nitrate tetrahydrate was also dissolved in deionized water to form a 1.67-mol/L solution. The mixture was stirred constantly for 2 h by a mechanical stirrer at 100°C. The pH was constantly adjusted and kept at 10 during the reaction. After the reaction, the deposited mixtures were washed several times with deionized water. The resulting material (HAp) was dried at 100°C for 72 h in an electrical air oven.

Silver-doped hydroxyapatite nanoparticles, Ca_10-*x*_Ag*_x_*(PO_4_)_6_(OH)_2_, with *x *= 0.2 (Ag:HAp), were performed by setting the atomic ratio of Ag/[Ag + Ca] at 20% and [Ca + Ag]/P as 1.67. The AgNO_3 _and Ca(NO_3_)_2 _· 4H_2_O were dissolved in deionized water to obtain 300 mL [Ca + Ag]-containing solution. On the other hand, the (NH_4_)_2_HPO_4 _was dissolved in deionized water to make 300 mL P-containing solution. The [Ca + Ag]-containing solution was put into a Berzelius and stirred at 100°C for 30 min. Meanwhile, the pH of P-containing solution was adjusted to 10 with NH_3 _and stirred continuously for 30 min. The P-containing solution was added drop-by-drop into the [Ca + Ag]-containing solution and stirred for 2 h and the pH was constantly adjusted and kept at 10 during the reaction. After the reaction, the deposited mixtures were washed several times with deionized water. The resulting material was dried at 100°C for 72 h.

### 2.2 Sample characterization

#### 2.2.1. XRD

The XRD was performed on a Bruker D8 Advance diffractometer, with nickel-filtered Cu Kμ (λ = 1.5418 Å) radiation, and a high efficiency one-dimensional detector (Lynx Eye type) operated in integration mode. The diffraction patterns were collected in the 2θ range 15°-140°, with a step size of 0.02° and 34 s measuring time per step. In an attempt to perform a complete XRD characterization of the nano-powders, the measured data were processed with the MAUD software, version 2.26 [33]. The instrumental line broadening has been evaluated using a heat-treated ceria powder proved to produce no observable size or strain line broadening.

#### 2.2.2. Scanning electron microscopy

The structure and morphology of the samples were studied using a HITACHI S2600N-type scanning electron microscope (SEM), operating at 25 kV in vacuum. The SEM studies were performed on powder samples. For the elemental analysis, the electron microscope was equipped with an energy dispersive X-ray attachment (EDAX/2001 device).

#### 2.2.4. TEM

TEM studies were carried out using a JEOL 200 CX. The specimen for TEM imaging was prepared from the particles suspension in deionized water. A drop of well-dispersed supernatant was placed on a carbon-coated 200 mesh copper grid, followed by drying the sample at ambient conditions before it is attached to the sample holder on the microscope.

#### 2.2.5. FT-IR spectroscopy

The functional groups present in the prepared powder and in the powders calcined at different temperatures were identified by FT-IR (Bruker Vertex 7 Spectrometer). For this, 1% of the powder was mixed and ground with 99% KBr. Tablets of 10 mm diameter for FTIR measurements were prepared by pressing the powder mixture at a load of 5 tons for 2 min and the spectrum was taken in the range of 400-4000 cm^-1 ^with resolution 4 and 128 times scanning.

#### 2.2.6. FT-Raman spectroscopy

Raman studies have been carried out at the wavelength excitation of 1064 nm using an FT Raman Bruker RFS 100 spectrophotometer. The laser was operated at 100 mW and up to 100 scans at 4 cm^-1 ^resolution were accumulated.

#### 2.2.7. XPS

Soft XPS is one of the most important techniques for the study of the elemental ratios in the surface region. The surface sensitivity (typically 40-100 Å) makes this technique ideal for measurements as oxidation states or biomaterials powder. In this analysis, we have used a VG ESCA 3 MK II XPS installation (*E*_*kα *_= 1486.7 eV). The vacuum analysis chamber pressure was *P ~ *3 × 10^-8 ^torr. The XPS recorded spectrum involved an energy window *w *= 20 eV with the resolution *R *= 50 eV with 256 recording channels. The XPS spectra were processed using Spectral Data Processor v 2.3 (SDP) software.

#### 2.2.8. In vitro antibacterial activity

The strains of bacteria used for this study were the strain of *Staphylococcus aureus *(ATCC 6538). The staphylococci were grown overnight in Todd-Hewit broth supplemented with 1% yeast extract at 37°C, followed by centrifuging. The supernatants were discarded and pellets were re-suspended in phosphate-buffered saline (PBS) followed by a second centrifuging and re-suspension in PBS. The samples to be tested were placed in 50 mL sterilized tubs followed by the addition of 2 mL of the bacterial suspension. The tubes were incubated at 37°C for 4 h. At the end of the incubation period, the samples were gently rinsed three times with PBS. The non-adherent bacteria were eliminated. After washing, the samples were then put into a new tube containing 5 mL PBS and vigorously vortexed for 30 s to remove the adhering microorganisms. The viable organisms in the buffer were quantified by plating serial dilutions on yeast extract agar plates. Yeast extract agar plates were incubated for 24 h at 37°C and the colony forming units were counted visually.

## 3. Results and discussions

**Figure 1:**
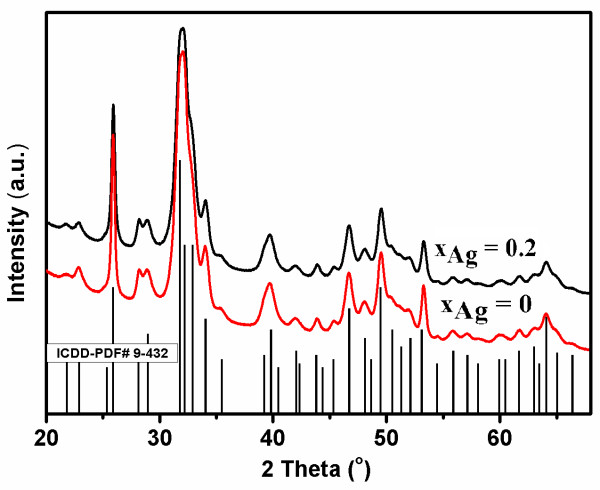
**Comparative representation of the experimental XRD patterns of the Ca_10-*x*_Ag*_x_*(PO_4_)_6_(OH)_2 _samples synthesized *x*Ag = 0 (HAp) and *x*Ag = 0.2 (Ag:HAp), and the characteristic lines of hydroxyapatite according to the ICDD-PDF number 9-432**.

The XRD patterns, presented in Figure 1, show the characteristic peaks of hydroxyapatite for each sample, according to ICDD-PDF no. 9-432, represented at the bottom of the figure, as reference. No other crystalline phases were detected beside this phase (Figure 1).

We performed whole powder pattern fitting by the Rietveld method of the as-prepared Ag-HAp structures. As a prerequisite for the atomic structure refinement, a good fit of the diffraction line profiles must be achieved. Because the peaks' broadening is related to the microstructural characteristics (crystallite size and microstrain) a suitable microstructure model is needed. Good pattern fit has been achieved using MAUD [33] for all the samples, by applying the Popa approach for the anisotropic microstructure analysis [34], implemented in the MAUD code as "Popa rules". It resulted that each sample is constituted of elongated nanocrystallites which can be approximated by circular ellipsoids, with the longer dimension parallel to the *c *crystallographic axis of HAp.

For the undoped HAp, Ag:HAp the length of the average crystallite (the average column size parallel to the *c*-axis) is around 43 nm and the width (the average column size perpendicular to the *c*-axis) is around 16 nm. The mean crystallite size averaged over all crystallographic directions is around 21 nm. For Ag:HAp, the length is around 38 nm and the width around 14 nm. The averaged diameter is around 19 nm.

The XRD of HAp and Ag:HAp also demonstrate that powders made by co-precipitation at 100°C exhibit the apatite characteristics with good crystal structure and no new phase or impurity is found.

**Figure 2:**
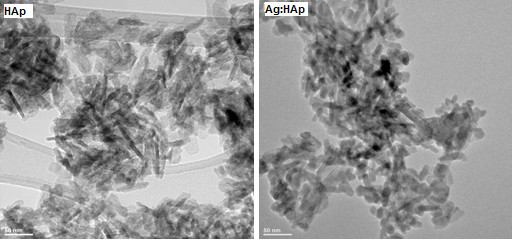
**TEM images of the Ca_10-*x*_Ag*_x_*(PO_4_)_6_(OH)_2 _samples with *x*Ag = 0 (HAp) and *x*Ag = 0.2 (Ag:HAp)**.

Figure 2 displays the TEM images of pure HAp (*x*Ag = 0) and Ag:HAp (*x*Ag = 0.2) with low resolution. Figure 2 (left) shows that HAp particles at 100°C are crystallized with a maximum size around 40 nm. In Figure 2 (right), the ellipsoidal-shaped Ag:HAp (*x*Ag = 0.2) particles about 30 nm are observed after Ca^2+ ^is partially substituted by Ag^+^. The substitution of Ca by Ag in the apatite structure leads to slight changes in the shapes of the nanoparticles. The morphology identifications indicated that the nanoparticles with good crystal structure could be made at 100°C by co-precipitation method.

**Figure 3:**
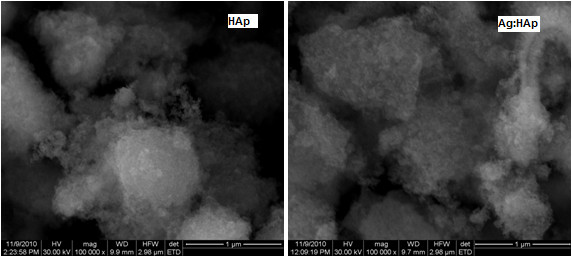
**SEM images of the Ca_10-*x*_Ag*_x_*(PO_4_)_6_(OH)_2 _samples with *x*Ag = 0 (HAp) and *x*Ag = 0.2 (Ag:HAp)**.

**Figure 4:**
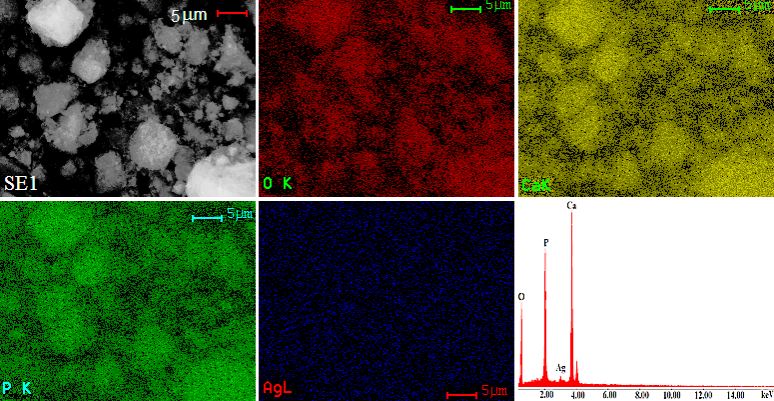
**EDAX spectrum of the Ag:HAp samples and simultaneous distributions of individual elements based on selected region of the sample**.

SEM (Figure 3) image and EDAX (Figure 4) spectrum of Ca_10-*x*_Ag*_x_*(PO_4_)_6_(OH)_2_, with *x *= 0 and 0.2, are shown. The morphology of the nanoparticles of HAp and Ag:HAp was investigated by SEM. SEM images provide the direct information about the size and typical shape of the as-prepared samples. The results suggest that the doping Ag^+ ^has little influence on the morphology of the HAp. The samples prepared at the atomic ratio Ag/[Ag + Ca] 20% (Ag:HAp) exhibit much smaller particle size. Elemental maps for the samples prepared at the atomic ratio Ag/[Ag + Ca] 20% are also shown. The spectrum and images confirmed the presence of silver on hydroxyapatite. The EDAX spectrum of Ag:HAp confirms the presence of calcium (Ca), phosphor (P), oxygen (O), and silver (Ag) in the samples.

**Figure 5:**
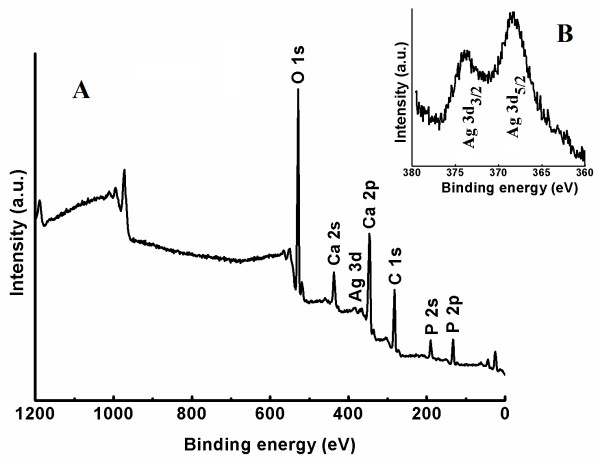
**XPS general spectrum of Ca_10-*x*_Ag*_x_*(PO_4_)_6_(OH)_2_, (*x*_Ag _= 0.2) powder (A). XPS narrow scan spectra for Ag (B)**.

XPS technique has been tested as a useful tool for qualitatively determining the surface components and composition of the samples. Figure 5 shows the survey XPS narrow scan spectra of Ag:HAp (*x *= 0.2) nanopowder obtained at 100°C and XPS narrow scan spectra of Ag element. In the XPS spectrum of Ag:HAp, the binding energy of Ca (2p, 347.3 eV), O (1s, 532.1 eV), and P (2p, 133.09 eV) can obviously be found (Figure 5A). The peaks of Ag (Ag(3d_5/2_) 368.4 eV and Ag((3d_3/2_) 374.3 eV) agree well with the literature [35]. XPS narrow scan spectra of Ag element are presented in Figure 5B. XPS results provide the additional evidence for the successful doping of Ag^+^, in Ag:HAp.

**Figure 6:**
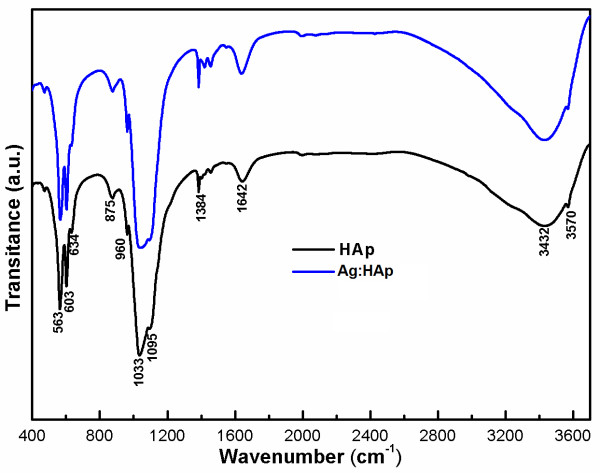
**Transmittance infrared spectra of the Ca_10-*x*_Ag*_x_*(PO_4_)_6_(OH)_2 _samples with *x*Ag = 0 (HAp) and *x*Ag = 0.2 (Ag:HAp)**.

FT-IR spectroscopy was performed to investigate the functional groups present in nanohydroxyapatite, Ca_10-*x*_Ag*_x_*(PO_4_)_6_(OH)_2_, with *x *= 0 and 0.2 obtained at 100°C by co-precipitation method (Figure 6). These data clearly revealed that the presence of the various vibrational modes corresponding to phosphates and hydroxyl groups. For all the samples, the presence of strong OH^- ^vibration peak could be noticed. The broad bands in the regions 1600-1700 and 3200-3600 cm^-1 ^correspond to H-O-H bands of lattice water [36-39]. The large bands which were attributed to adsorbed water diminished for the HAp_Ag20 sample. The changes are attributed to the substitution of Ag^+ ^from Ca^2+ ^into the lattice of apatite.

Bands' characteristics of the phosphate and hydrogen phosphate groups in apatitic environment were observed: 563, 634, 603, 960, and 1000-1100 cm^-1 ^for the PO_4_^3- ^groups [39,40] and at 875 cm^-1 ^for the HPO_4_^2- ^ions [41]. Moreover, it should be noted that the HPO_4_^2- ^band was present in all the spectra but for high values of Ag/(Ca+Ag) atomic ratio the band diminished. The small CO^2- ^band was presented in the spectra with atomic ratio Ag/(Ca + Ag) = 20% at 1384 cm^-1 ^[41].

**Figure 7:**
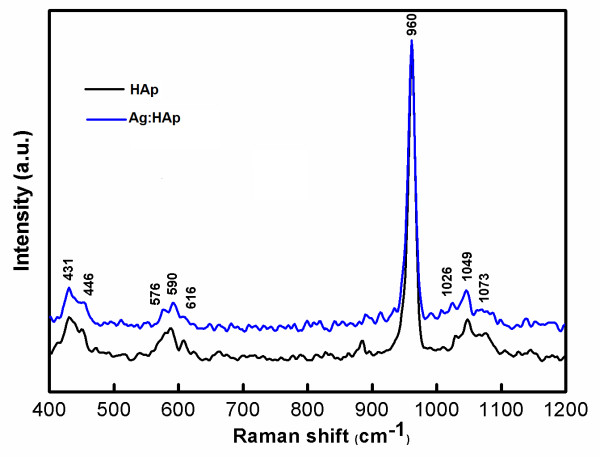
**FT-Raman spectra of the Ca_10-*x*_Ag*_x_*(PO_4_)_6_(OH)_2 _samples with *x *= 0 (HAp) and *x *= 0.2 (Ag:HAp)**.

Complementary information can be obtained from FT-Raman spectroscopy (Figure 7). The internal modes of the PO_4_^3- ^tetrahedral ν_1 _frequency (960 cm^-1^) corresponds to the symmetric stretching of P-O bonds. The vibrational bands at 429 cm^-1 ^(ν_2_), 450 cm^-1 ^(ν_2_) are attributed to the O-P-O bending modes. We assigned the bands present at 1046 cm^-1 ^(ν_3_) and 1074 cm^-1 ^(ν_3_) to asymmetric ν_3 _(P-O) stretching. The ν_4 _frequency (589 and 608 cm^-1^) can be addressed mainly to O-P-O bending character [42].

Bands observed in the FT-IR and FT-Raman spectroscopies are characteristic of crystallized apatite phase. However, the intensity of vibration peak decreases when the atomic ratio Ag/(Ca + Ag) is 20%. These results are in agreement with the XRD patterns, evidencing the crystallized apatitic phase and the apatitic phase is the only one detected.

**Figure 8:**
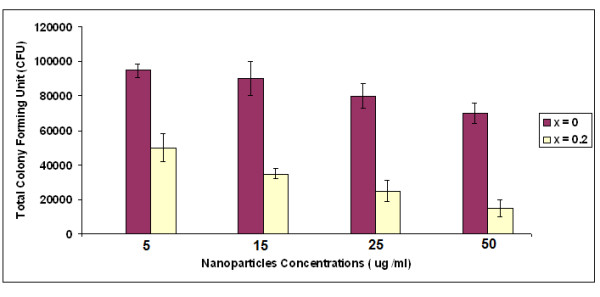
**Adherence of *Staphylococcus aureus *on different concentrations of Ca_10-*x*_Ag*_x_*(PO_4_)_6_(OH)_2 _(*x *= 0 and 0.2) nanopowders**.

Figure 8 shows the results of viable bacteria adhering to the 5, 15, 25, and 50 μg/mL of Ca_10-*x*_Ag*_x_*(PO_4_)_6_(OH)_2_, (*x *= 0 and 0.2) when exposed to *Staphylococcus aureus*. Bacterial adhesion were significantly reduced on sample with *x *= 0.2 when compared to samples with *x *= 0. However, no significantly difference in *Staphylococcus aureus *adhesion was observed between the different concentration of Ag:HAp nanopowder.

Significant differences in bacterial adhesion on HAp (*x *= 0) and Ag:HAp (*x *= 0.2) were observed. The Ag:HAp nanopowders were observed to have significantly lower adhesion of *Staphylococcus aureus*, suggesting that the Ag:HAp nanopowders were antibacterial. In the future, the effect of silver-doped hydroxyapatite on other bacteria strains will be evaluated and these strains will be selected depending on the field of applications. The influence of atomic ratio Ag/[Ca + Ag] on bacteria strains will be also studied.

## 4. Conclusions

In this article, we have described an easy simple and low-cost method for obtaining a Ag:HAp nanoparticles powders. Nanocrystalline antibacterial Ag:HAp with *x*Ag from 0 (HAp) to 0.2 (Ag:HAp) can be made at 100°C by co-precipitation. The Ag^+ ^partially substitutes for calcium and enters the structure of hydroxyapatite.

The XRD studies have shown that the characteristic peaks of hydroxyapatite in each are presented. The Popa model for size and microstrain anisotropy used in this article is a reliable method for crystallite size and microstrain measurement. The morphology identifications by TEM indicated that the nanoparticles with good crystal structure could be made at 100°C by co-precipitation method.

In the agreement with the results of XRD and TEM, the FTIR and FT-Raman spectra of the HAp show the absorption bands characteristic of hydroxyapatite. XPS results provide the additional evidence for the successful doping of Ag^+^, in Ag:HAp.

The inhibition of bacteria containing different concentrations of HAp (*x *= 0) and Ag:Hap (*x *= 0.2) nanopowders was investigated in *Staphylococcus aureus*. The Ag:HAp nanopowders show strong antibacterial activity. *In vitro *bacterial adhesion study indicated a significantly reduced number of *Staphylococcus aureus *on different concentrations of Ag:Hap (*x *= 0.2) nanopowders. In conclusion, we have demonstrated a highly facile and simple methodology for preparing silver-doped hydroxyapatite nanopowder.

## Abbreviations

EDAX: energy-dispersive X-ray spectroscopy; FT-IR spectroscopy: Fourier transform infrared spectroscopy; FT-Raman spectroscopy: Fourier transforms Raman spectroscopy; SEM: scanning electron microscopy; TEM: transmission electron microscopy; XRD: X-ray diffraction.

## Competing interests

The authors declare that they have no competing interests.

## Authors' contributions

CSC and DP conceived the study. CSC, LVS, and DP performed the synthesis of the powders. Characterization of materials was carried out by FM, CSC, and DP. DP directed the study and wrote the draft paper. All authors contributed to the interpretation of results, discussion and read, corrected and approved the final manuscript.
